# The Role of Slurry Reflux in a Corn Stalk Continuous Anaerobic Digestion System: Performance and Microbial Community

**DOI:** 10.3390/ijerph20031687

**Published:** 2023-01-17

**Authors:** Ling Zhao, Yang Gao, Jiaxing Sun, Yanan Wang, Congxin Wang, Shuai Yu, Zhen Wang, Jingyang Li, Ronghou Liu, Wei Kou

**Affiliations:** 1 College of Engineering, Shenyang Agricultural University, Shenyang 110866, China; 2 Biomass Energy Engineering Research Centre, School of Agriculture and Biology, Shanghai Jiao Tong University, 800 Dongchuan Road, Shanghai 200240, China; 3 Department of Chemistry and Environmental Engineering, Yingkou Institute of Technology, Yingkou 115014, China

**Keywords:** slurry reflux, corn stalk, anaerobic digestion, methane, microbial community

## Abstract

Slurry reflux is a low-cost slurry reduction technology, which can solve the problem that a large amount of slurry cannot be completely consumed in a biogas plant. Anaerobic digestion (AD) of corn stalks with slurry reflux and non-reflux was compared and evaluated in continuous anaerobic digestion to clarify the effects of slurry reflux on AD with organic loading rate (OLR) variation. It was found that slurry reflux increased cumulative methane production and improved system stability. The average methane yield of the slurry reflux group was 224.19 mL/gVS, which was 41.35% higher than that of the non-reflux group. High-throughput sequencing results showed that slurry reflux increased the microbial community richness. The dominant microorganisms in the reflux group were in phylum Bacteroidetes, which have the capacity to degrade polymers, and *Methanothrix*, which is an aceticlastic methanogen. The relative abundances of Bacteroidetes and *Methanothrix* were 32.41% and 41.75%, respectively. *Clostridium* III and *Saccharofermentans*, which are related to syntrophic acetate oxidation and hydrolysis, were increased in relative abundance in the slurry reflux system. The increase of the OLR altered the main methane-producing pathway from the acetoclastic methanogenic pathway to the hydrogenotrophic methanogenic pathway in the AD system, and the slurry reflux can delay this trend. This study provided an effective way for the reduction and utilization of slurry in a biogas plant.

## 1. Introduction

China is rich in crop stalk resources, with an annual output of approximately 850 million tons [1]. However, the utilization rate of these resources has been relatively low [2]. Massive waste of resources and serious air pollution pose a great threat to the construction of ecological civilization in rural areas, which is a social and environmental problem that China needs to solve urgently [3]. Of the various methods for dealing with organic waste, anaerobic digestion (AD) is the most promising [4]. As an engineering solution to degrade organic waste and produce biogas, AD was considered as a sustainable and effective approach to deal with organic waste, generated clean energy, and recycled organic matter by utilizing crop stalks [5]. With the development of large-scale biogas projects, the original fermentation material is gradually becoming not enough, thus, sustainable material like crop stalk material should be emphasized [6,7].

However, the production of methane by AD also produces an amount of slurry, which is rich in nitrogen, phosphorus, potassium, and other nutrients [8]. The remaining slurry, called digestate, which can be around 90–95% of what was fed into the AD process, is a nutrient-rich by-product [9]. The slurry would inevitably cause secondary pollution, if it was not properly utilized [10,11]. Slurry reflux is therefore an efficient and inexpensive way to reduce emissions from the AD system, which in turn, can effectively reduce the slurry discharge and the subsequent disposal cost [12]. The basicity of the slurry can be used to balance the acidity of the digestion liquid through the slurry reflux and maintain the pH value within an appropriate range. Yanjing et al. reported that digestate recirculation could promote the methane production rate in the co-digestion continuous AD system of food waste and cow dung, and both co-digestion and digestate recirculation technology could maintain the pH in the system [4]. Zhuang et al. reported that effluent recirculation showed a considerable positive effect in alleviating volatile fatty acids (VFAs) inhibition and improving biogas production in the AD reactor because of the effect of dilution and pH adjustment [13]. However, these studies were conducted on a two-stage AD system, which increased costs and energy consumption compared with a single stage AD system.

The AD system with stalk as the substrate was not as stable as that with manure as the substrate, because of the lack of nutrients and buffering capacity [14]. Additionally, slurry reflux can improve the richness and vitality of microorganisms in the AD system; it is beneficial to the AD system using crop stalks as the raw material [15,16].

The biogas-production efficiency and system stability of the AD system depends largely on the activity of functional microorganisms, while the diversity and richness of the microbial community can also affect its performance [17]. In the AD system, the stages of acid production, acetic acid production, and methane production are completed by different microbial communities. By studying the community structure of bacteria and archaea in the AD system, the internal relationships and interaction between functional microorganisms and the AD performance can be obtained [18,19]. Li et al. used corn stalks as a substrate with slurry reflux and found that the serial system was enriched in species of *Bacteroidetes* and *Firmicutes* [20]. The microbial community can not only provide an early warning of system stability but also reveal mechanisms of the AD system [18,19]. Besides, different substrates determine the species and abundance of microbial communities in the system, which would also lead to differences in various enzymes present [19].

However, whilst recirculation can improve fermentation efficiency and system stability by recycling nutrients, buffers, and microorganisms [21], it can also result in the accumulation of fermentation inhibitors and non-degradable substances leading to inefficient digestion and even process failure [22].

Currently, there is a lack of research on the continuous single stage AD of corn stalks with full slurry reflux, especially the relationship between microbial communities in the AD system with the gradually increase of OLR. Therefore, this study examined the system stability and the influence of the microbial community, setting up an AD system, which incorporated slurry reflux. Corn stalks were used as the substrate, and various OLR were set, that aimed to provide a scientific basis for the application of slurry reflux for the practical project.

## 2. Materials and Methods

### 2.1. Substrates and Inoculum

Corn stalk was collected from the Beishan Scientific Research Base of Shenyang Agricultural University and crushed to 2–3 mm after natural drying. The inoculum was obtained from the AD pond, which used pig measure as feedstock, in the Comprehensive Energy Demonstration Base of Shenyang Agricultural University. The physicochemical parameters of the corn stalk and inoculum are shown in Table 1. The total solids (TS) of corn stalk and inoculum were 90.23% and 17.46%, while the volatile solid (VS) of these two substrates were 84.62% and 6.12%, respectively.

### 2.2. Experimental Digester and Setup

**Figure 1:**
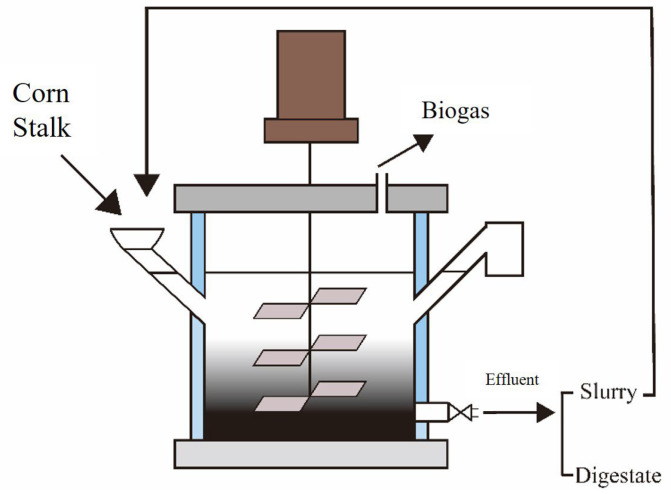
Schematic diagram of the CSTR.

All the AD experiments were performed in two continuous stirred tank reactors (CSTR) with a total volume of 10 L (working volume was 8 L) (Figure 1). The temperature was maintained at 37 ± 1 °C, the TS content was 10%, and the mixing ratio of inoculum and substrate was 1:3. The slurry mixing was carried out once every 3 d. The slurry was mixed with corn stalk as the reflux to the reflux group (R2), while tap water was used instead of the slurry in non-reflux group (R1). The stirring was set for 20 min every 2 h, and the rotating speed was 30 rpm. The above continuous anaerobic reactor experiments were based on previous laboratory research results [23]. The first 10 days of the process comprise the start-up period (no data). Slurry reflux started on day 11. Three hydraulic retention periods were conducted in the continuous experiment. According to the different OLR, the test was divided into three phases: Phase Ⅰ (11–37 d), Phase Ⅱ (38–64 d) and Phase Ⅲ (65–91 d) with the OLR 2.0 gTS/(Lreactor·d), 3.0 gTS/(Lreactor·d) and 4.0 gTS/(Lreactor·d), respectively. The biogas volume and composition were simultaneously analyzed every day, and the slurry samples were taken every 3 days for further analysis.

### 2.3. Analytical Techniques and Statistical Method

The pH value, TS and VS concentrations were measured according to the Standard Methods [24]. The oxidation-reduction potential (ORP) was measured using a Hach HQ40D multi-parameter water quality analyzer. The biogas volumes were monitored using the water displacement method and then converted to a volume at standard conditions. A portable biogas analyzer (GA2000, Geotech, Norfolk, UK) was used for proportional analysis of the biogas. The VFA concentrations were determined using gas chromatography on a FID detector, 19091N-133 (30 m × 250 μm × 0.26 μm) column. Ammonia nitrogen was used for determination in the sodium reagent colorimetric method. Total inorganic carbon (TIC) was determined by the Elab-TOC analyzer.

From the two groups of AD reactors with varying conditions, a total of six samples were selected for microbial community structure analysis, these samples represented pre-reflux material (R1-1; R2-1), the end of Phase Ⅰ (R2-2), the end of Phase Ⅱ (R1-3; R2-3), and the end of Phase Ⅲ (R2-4).

The extraction of sample DNA was conducted following the instructions of the OMEGA E.Z.N.ATM Mag-Bind Soil DNA Kit. The diluted DNA samples were amplified using PCR (T100TM Thermal Cycler, Bio-Rad, Hemel Hempstead, UK). Microbial amplification primers were 341F: 5′-CCCTACACGACGCTCTTCCGA TCTG (BARCODE) CCTACGGGNGGCWGCAG-3′ and 805R: 5′-GACTGGAGTTCCTTGGCACCCGAGAA TTCCAGACTACHVGGGTA TCTAA TCC-3′. Archaea expansion increased 349 f: primers for 5′- CCCTACACGACGCTCTTCCGA TCTN (barcode) GYGCASCAGKCGMGAAW-3 and 806 r:’ 5-’GACTGGAGTTCCTTG-GCACCCGAGAA TTCCAGGACTACVSGGGTA TCTAA T-3′. The PCR products with normal amplified fragments of bacteria and archaea above 400 bp were treated with 0.6 magnetic beads (Agencourt AMPure XP, Beckman Coulter, Brea, CA, USA). After treatment, the samples were sent to Shanghai Sangon Bioengineering Co., Ltd. (Shanghai, China) for sequencing. The miseq sequencing data has been uploaded to NCBI (https://www.ncbi.nlm.nih.gov/, accessed on 1 January 2023), and the BioProject ID is PRJNA772525, SRA submission is SUB10523577. The detection types were bacteria and archaea, the platform was Miseq2 × 300 bp, and the library was the NCBI16S database. Operational taxonomic unit (OTU) clustering and species annotation were performed for more than 97% of the sequences, and Alpha diversity analysis and relative abundance analysis were also performed.

## 3. Results

### 3.1. Influence of Slurry Reflux on Methane Production

Methane production rate and cumulative methane production were important indicators to measure the performance of the AD system. The average methane production rate and cumulative methane production at each phase in AD process are shown in Table 2. 

In the AD system, OLR ranged from 2 to 4 gTS/(Lreactor·d), and the average methane production rate showed a decreasing trend, from 202.35 and 260.25 to 118.70 mL/gVS and 202.36 mL/gVS, respectively. It was found that slurry reflux increased methane yield. The average methane yield of slurry reflux group was 224.19 mL/gVS, which was 41.35% higher than that of the non-reflux group. The average volumetric methane production rate increased significantly from 1.03 and 1.32 to 1.20 and 2.05 L/(Lreactor.d), respectively. At the same time, the cumulative methane production in each phase also gradually increased, which in R1 and R2 were 74.00 L, 84.88 L and 86.76 L, and 95.10 L, 115.12 L and 147.92 L, respectively. The cumulative methane production of R2 increased by 28.6%, 35.6% and 70.5%, compared with R1 in three phases, respectively. The results showed that slurry reflux can bring the organic matter that has not been degraded back into the fermentation system and reduce the discharge of microorganisms, which can improve the methane yield. High organic loading is unfavorable to the methane conversion of the substrate [25]. With the increase in organic loading, the methane yield showed a downward trend, but the slurry reflux could act as a buffer to slow down the trend.

### 3.2. Influence of Slurry Reflux on System Stability

**Figure 2:**
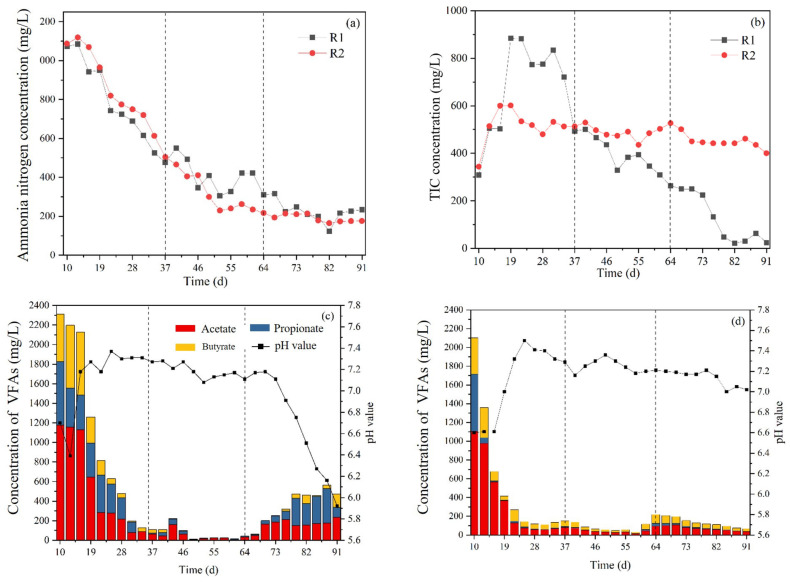

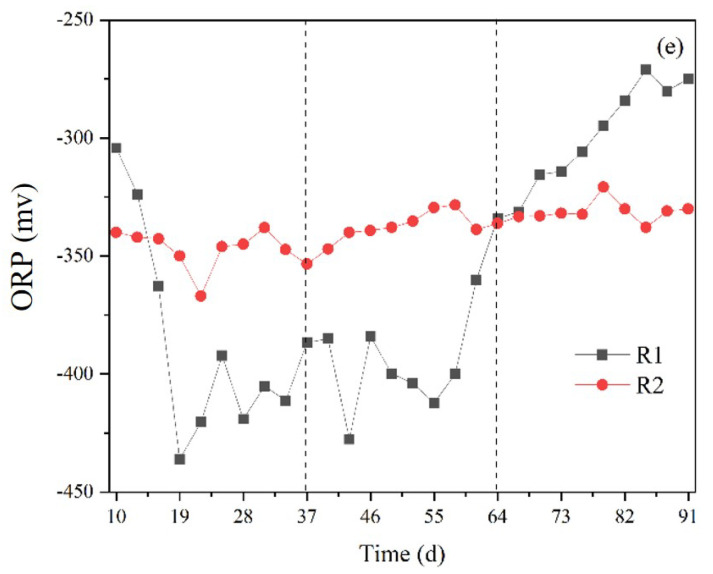
Concentration of ammonia nitrogen (**a**); concentration of total inorganic carbon (**b**); concentration of volatile fatty acids and pH value of R1 (**c**) and R2 (**d**); ORP (**e**); in Phase I–Phase III;, respectively, had organic load rates of 2, 3, and 4 gTS/(Lreactor·d).

Ammonia nitrogen concentration, inorganic carbon concentration, VFAs, pH value and ORP were the key factors of system stability [26]. The variation indirectly reflected the stability of the AD system (Figure 2).

The concentration of ammonia nitrogen decreased gradually from ~1100 mg/L to ~200 mg/L during the whole AD process. With the increase of OLR, the TIC concentration of R1 decreased from 883.45 mg/L to 24.06 mg/L; R2 decreased slowly but remained at 500 ± 100 mg/L. A stable TIC environment was very important for the stability of corn stalk anaerobic digestion [27]. The variation trend of VFAs concentration was similar to that of ammonia nitrogen, which decreased significantly, from 2310.99 mg/L and 2101.90 mg/L to 14.5 and 29.1 mg/L, respectively. At the end of phase Ⅱ, the VFAs concentrations in both groups were low. The VFAs concentration of R1 increased, when OLR was 4 gTS/(Lreactor·d), and pH value dropped to 5.92, which inhibited methanogens metabolism. The peak pH values of R2 decreased gradually from 7.50 to 7.02. The ORP of R1 and R2 increased, when OLR increased, and the variation of R1 was more significant from −436.10 to −271.70 mV, and the average values of R2 in three phases, which were maintained in a suitable range, were −347.90 mV, −336.90 mV and −331.10 mV, respectively. With the increase of OLR, the pH value and oxidation-reduction potential (ORP) of the reflux group remained 7.16 ± 0.23 and −338.71 mV ± 9.22, indicating that the system was relatively stable.

### 3.3. Microbiological Analysis during Biogas Production Process

#### 3.3.1. Dynamics of Microbial Community Richness and Diversity

The microbial diversity parameters, including Shannon, Simpson, Ace, and Chao 1, estimated at different phases of the AD systems are shown in Table 3. 

The bacterial community diversity of the reflux group and the non-reflux system showed a downward trend, and decreased by 1.1% and 6.4%, respectively. The diversity of archaea increased by 39% and 20%, respectively. With the increase of OLR, the bacterial community richness of R2 increased from 1414.02 to 1543.50, and the archaea community richness increased from 211.22 to 277.54. The results verified that slurry reflux increased the richness of microbial community, which was a successful manifestation of the biochemical activities of the anaerobic system [28,29].

##### Dynamics of Bacterial Populations

**Figure 3:**
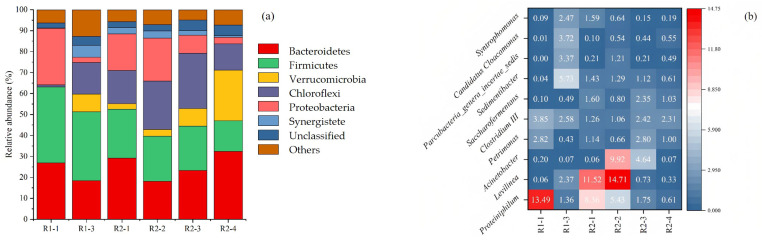
Bacterial sequence distributions: (**a**) Percent of bacterial community abundance at the phylum level; (**b**) Percent of bacterial community heatmap at the genus level.

The relative abundance of bacterial communities at phylum level in samples at different phase is shown in Figure 3a. There were six dominant phyla found in this study. They were Bacteroidetes, Firmicutes, Proteobacteria, Chloroflexi, Verrucomicrobia, and Synergistetes. The dominant phyla accounted for 83.14–91.75% of the total. Firmicutes and Bacteroidetes were dominant in the two groups, and their relative abundance accounted for 39.55–51.29%. During the AD process, the relative abundances of Firmicutes and Bacteroidetes were negatively correlated, the relative abundance of Bacteroidetes was from 18.07% (R2-2) to 32.41% (R2-4), Firmicutes decreased from 21.48% to 14.57%. Yuan et al. found that Firmicutes was the dominant in the unstable start-up stage of the AD system, which rapidly declined after the system stabilized, and Bacteroidetes became the dominant during the stability of the system [30].

The relative abundance of Proteobacteria decreased when OLR increased from 2 gTS/(Lreactor·d) to 4 gTS/(Lreactor·d). The peak value of Verrucomicrobia was 25.84% when OLR was 4 gTS/(Lreactor·d), which increased 20.84% compared with 2 gTS/(Lreactor·d). The relative abundances of Chloroflexi under different OLR were 23.36%, 29.52% and 13.52%, respectively, which peaked at 3 gTS/(Lreactor·d). The relative abundance of each phylum changed significantly, indicating that the bacterial community structure was significantly affected by OLR [31]. 

The heatmap of bacterial communities at genus level in samples at different phases is shown in Figure 3b. The relative abundances of the four genera *Candidatus Cloacamonas*, *Syntrophomonas*, *Sedimentibacter* and *Parcubacteria* genera incertae sedis in R1, which were higher than R2 at OLR was 3.0 gTS/(L.d), were 3.72%; 2.47%; 5.73% and 3.37%, respectively.

The relative abundances of *Proteiniphilum*, *Levilinea* and *Acinetobacter* in R2 were 5.43%, 14.71% and 9.92% when the OLR was 2.0 gTS/(L.d), and decreased to 1.75%, 0.73% and 4.64% when OLR increased to 4.0 gTS/(L.d), respectively. *Clostridium* III was enhanced as OLR increased from 1.06 to 2.31, *Petrimonas* and *Saccharofermentans* also had a peak when the OLR was 3.0 gTS/(L.d). *Clostridium* III and *Saccharofermentans*, which were related to syntrophic acetate oxidation and hydrolysis, were increased in relative abundance in the slurry reflux system. 

##### Dynamics of Archaeal Populations at the Phylum and Genus Level

**Figure 4:**
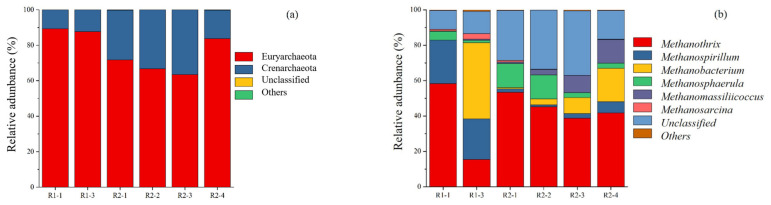
Variation of archaeal community structure; (**a**) Percent of archaeal community abundance at phylum level; (**b**) Percent of archaeal community abundance at genus level.

Only 1% of the archaea in the system belonged to unknown phylum, and there were almost no unknown archaea taxa in the AD system. Archaea at the phylum level (Figure 4a) were dominated by microorganisms from two groups, Euryarchaeota and Crenarchaeota; the relative abundance of Euryarchaeota in the different OLR were over 60%, and the second dominant was Crenarchaeota.

At the genus level (Figure 4b), the dominant microorganisms included *Methanothrix*, *Methanospirillum*, *Methanobacterium*, *Methanosphaerula* and *Methanomassiliicoccus*. The relative abundances of *Methanothrix* at different OLR (R2-2, R2-3, R2-4) were, 45.16%, 38.76% and 41.75%, respectively. In the non-reflux group (R1-1, R1-3), the relative abundances were 58.24%, 15.34%, respectively.

*Methanospirillum*, *Methanobacterium*, *Methanosphaerula* and *Methanomassiliicoccus* are hydrogenotrophic methanogens [32,33,34,35]. With the increase of OLR, the relative abundances were 17.17%, 21.10%, 24.14% and 41.39%, respectively, showing an upward trend. Compared with R1 (R1-3) and R2 (R2-3), hydrogenotrophic methanogens in the non-reflux group (R1-3) were overwhelmingly dominant, with a relative abundance of 68.05%, higher than hydrogenotrophic methanogens in R2 (R2-3) under the same OLR, which was 43.19%. When OLR increased to 4 gTS/(Lreactor·d), the relative abundance of hydrogenotrophic methanogens in R2 increased to 41.39%, which was similar to that of *Methanothrix* (41.75%). The increase of OLR altered the main methane-producing pathway from the acetoclastic methanogenic pathway to the hydrogenotrophic methanogenic pathway in the AD system, and slurry reflux can delay this trend. 

## 4. Discussion

Methane production rate in the AD system decreased with the increase of OLR, which could be effectively alleviated by slurry reflux. Methane production rate of R1 and R2 decreased by 41.34% and 22.24%, when OLR was 2 to 4 gTS/(Lreactor·d), respectively. With the increase of OLR, the volumetric methane production rate is increased, and the slurry reflux has a significant promoting effect on the volumetric methane production rate. Average volumetric methane production rate was an index reflecting reactor efficiency. Slurry reflux carried undegraded organic matter back to the AD system and increased methane production, which was consistent with a previous report [20]. Although the substrate used with this study was corn stalk, compared with the previous study [20], as far as we know, there was a lack of discussions about AD systems operating at higher OLR. In large-scale projects, average volumetric methane production rate was also an important index. The average volumetric methane production rates, which were in this research 1.32, 1.60 and 2.05 L/(Lreactor.d), were higher than in previous studies. A higher average volumetric methane production rate can improve the volume utilization rate of reactor and single-phase AD and would also reduce the operating cost. Besides, Han et al. [36] and Zhang et al. [37] found that reflux inhibited AD and reduced methane production, which was mainly caused by nitrogen deficiency in the long-term AD processing of corn stalk. In this test, an 81-d slurry reflux operation did not show a significant decrease in methane production.

The concentration of VFAs and ammonia nitrogen were high at the initial stage, which might be caused by the short start-up phase. In this experiment, only a 10-d start-up phase was set. Ammonia was an essential nutrient for anaerobic microbes [38]. However, corn stalks were high carbon substrates, the ammonia nitrogen was gradually decrease with the system operation time [31]. As CO_2_ absorption rates increase with increasing pH, when pH decreased, the TIC in the liquid phase decreased.

Bacteria had higher microbial population richness and diversity than archaea in the AD system, which was mainly caused by the diversity difference of bacteria and archaea [39]. The diversity and richness of bacterial and archaea communities were correlated with the stability of the system. The relative abundance of Firmicutes, which exhibit high hydrolytic and acidogenic activities, in R1 was higher than that in R2, which supported VSAs accumulation achieved in R1. Bacteroidetes established synergic metabolism with acetoclastic methanogens for biogas production, which had various functions in the AD process, and became the dominant bacterial community, indicating that slurry reflux can improve the stability of the AD system [30]. At genus level, the relative abundances of *Proteiniphilum*, *Levilinea*, *Acinetobacter* and *Candidatus Cloacamonas*, which are acidogens, decreased as OLR increasing [40]. *Syntrophomonas*, a typical syntrophic bacteria for fatty acid oxidization, and *Clostridium* III, a syntrophic acetate oxidation bacteria (SAOB), were higher than R2(R2-3), because the hydrotrophic methanogens, in R1 when OLR was 3.0 gTS/(L.d), were higher [36]. *Saccharofermentans*, a cellulolytic genus, had higher relative abundance in R2 [41]. Besides, there was still to account the unclassified bacteria at the genus level, especially in R2-4, which was 75.5% when OLR was 4.0 gTS/(L.d), and therefore needs further study.

Euryarchaeota were the dominant archaean microorganisms at phylum level, a result that is supported by some previous research [20,42]. The relative abundance of *Methanothrix* in the reflux group (R2-3) was higher than that in non-reflux group (R1-3), which may be due to the continuous outflow of slurry in the non-reflux group and that the VFAs concentration was low. The relative abundance of hydrotrophic methanogens in R2 increased when OLR increased. This was because hydrotrophic methanogens were more dominant in AD systems under higher OLR [43].

## 5. Conclusions

Slurry reflux improved the performance of the AD system, reduced slurry emission and improved the methane production rate. The stability of the AD system was significantly improved by slurry reflux, and pH and ORP values were maintained in the appropriate range. The results of high-throughput sequencing analysis showed that slurry reflux slowed down the decline of microbial community diversity and increased the richness of the bacterial community. The dominant microorganisms were Bacteroidetes and *Methanothrix* in the reflux group. The relative abundance of *Clostridium* III and *Saccharofermentans*, related to acetic acid intercamp oxidation hydrolysis, was improved in the slurry reflux system. The increase of OLR altered the main methane-producing pathway of the AD system and slurry reflux could delay it.

## Figures and Tables

**Table 1:** Physicochemical properties of corn stalks and inoculum.

Parameters	Corn Stalk	Inoculum
TS (%*w*/*w*)	90.23 ± 0.07	17.46 ± 0.12
VS (%*w*/*w*)	84.62 ± 0.04	6.12 ± 0.08
C (%*w*/*w*)	42.75 ± 0.21	11.32 ± 0.14
N (%*w*/*w*)	0.75 ± 0.07	0.77 ± 0.09
pH	-	7.60 ± 0.1

**Table 2:** Methane production performance at each phase in AD process.

Parameters	Phase Ⅰ		Phase Ⅱ		Phase Ⅲ	
	R1	R2	R1	R2	R1	R2
Time (d)	11–37		38–64		65–91	
Organic load rate (gTS/(Lreactor·d))	2.0	2.0	3.0	3.0	4.0	4.0
Average methane production rate (mL/gVS)	202.35	260.25	154.80	209.95	118.70	202.36
Average volumetric methane production rate (L/(Lreactor.d))	1.03	1.32	1.18	1.60	1.20	2.05
Cumulative methane production (L)	74.00	95.10	84.88	115.12	86.76	147.92

**Table 3:** Alpha diversity parameters of the microbial community during the anaerobic digestion process.

	Groups	R1-1	R1-3	R2-1	R2-2	R2-3	R2-4
Bacterial	Seq num	50,509	58,538	55,398	43,665	22,254	40,155
	OTUs	1227	1133	1284	1105	1116	1354
	Ace	1494.53	1386.81	1468.03	1434.84	1992.30	1705.42
	Shannon	4.59	4.54	4.55	4.55	4.26	3.98
	Chao1	1406.35	1332.23	1414.02	1357.67	1514.35	1543.50
Archaea	Seq num	35,502	72,156	66,784	79,634	59,710	66,585
	OTUs	141	219	150	159	108	212
	Ace	271.08	420.89	294.22	234.75	273.60	356.38
	Shannon	1.59	2.21	1.63	1.92	1.95	2.02
	Chao1	220.50	304.00	211.22	209.32	174.00	277.54

## Data Availability

Not applicable.

## References

[1] Li K., Liu R., Sun C. (2016). A review of methane production from agricultural residues in China. Renew. Sustain. Energy Rev..

[2] Du H., Zhang Y.X., Zhang X.Y., He Z., Mao W.W. (2017). Bibliometric analysis on the research status and development of straw utilization in China. J. Liaoning Univ. Nat. Sci. Ed..

[3] Liu C., He Z., Lu X. (2022). Optimization analysis of carbon emission reduction from crop straw collection and transportation under the sustainable development goals. Trans. CSAE.

[4] Lee M., Hidaka T., Hagiwara W., Tsuno H. (2009). Comparative performance and microbial diversity of hyperthermophilic and thermophilic co-digestion of kitchen garbage and excess sludge. Bioresour. Technol..

[5] Yanjin W., Zhenfeng W., Quanguo Z., Gaoshen L., Chenxi X. (2020). Comparison of bio-hydrogen and bio-methane production performance in continuous two-phase anaerobic fermentation system between co-digestion and digestate recirculation. Bioresour. Technol..

[6] Yu Q., Liu R., Li K., Ma R. (2019). A review of crop straw pretreatment methods for biogas production by anaerobic digestion in China. Renew. Sustain. Energy Rev..

[7] Rubén A., José M.L., Manoj K., Mohammad A.S., Muhammad U.K., Abid S., Muhammad S., Maksim R., Muhammad U. (2022). Anaerobic Digestion of Lignocellulose Components: Challenges and Novel Approaches. Energies.

[8] Lu J., Zhu L., Hu G., Wu J. (2010). Integrating animal manure-based bioenergy production with invasive species control: A case study at Tongren Pig Farm in China. Biomass Bioenergy.

[9] Lamolinara B., Pérez-Martínez A., Guardado-Yordi E., Fiallos C.G., Diéguez-Santana K., Ruiz-Mercado G.J. (2022). Anaerobic digestate management, environmental impacts, and techno-economic challenges. Waste Manag..

[10] Chen C., Ruan Z.Y., Wu J., Gao L.H., Song J.L., Wang Y.W., Xu Y.S., Wei X.L., Xu F.H. (2013). Research Progress on the Comprehensive Disposal and Utilization of Biogas Slurry from Large Scale Biogas Engineering. China Biogas.

[11] Qiu S., Zhao L.B., Sun Y. (2016). The influence of aeration rate on intermittent forced-aeration composting of biogas residue. China Environ. Sci..

[12] Wu C., Huang Q., Yu M., Ren Y., Wang Q., Sakai K. (2018). Effects of digestate recirculation on a two-stage anaerobic digestion system, particularly focusing on metabolite correlation analysis. Bioresour. Technol..

[13] Zhuang Z., Wu S., Zhang W., Dong R. (2013). Effects of organic loading rate and effluent recirculation on the performance of two-stage anaerobic digestion of vegetable waste. Bioresour. Technol..

[14] Zheng Z., Cai Y., Zhao Y., Meng X., Zhang Y., Lu C., Hu Y., Cui Z., Wang X. (2020). Achieve clean and efficient biomethane production by matching between digestate recirculation and straw-to-manure feeding ratios. J. Clean. Prod..

[15] Hu Y., Shen F., Yuan H., Zou D., Pang Y., Liu Y., Zhu B., Chufo W.A., Jaffar M., Li X. (2014). Influence of recirculation of liquid fraction of the digestate (LFD) on maize stover anaerobic digestion. Biosyst. Eng..

[16] Yang Q., Ju M.T., Li W.Z. (2016). Review of methane production from straws anaerobic digestion. Trans. CSAE.

[17] Razaviarani V., Buchanan I.D. (2015). Anaerobic co-digestion of biodiesel waste glycerin with municipal wastewater sludge: Microbial community structure dynamics and reactor performance. Bioresour. Technol..

[18] Abbassi-Guendouz A., Brockmann D., Trably E., Dumas C., Delgenès J.-P., Steyer J.-P., Escudié R. (2012). Total solids content drives high solid anaerobic digestion via mass transfer limitation. Bioresour. Technol..

[19] Rui J., Li J., Zhang S., Yan X., Wang Y., Li X. (2015). The core populations and co-occurrence patterns of prokaryotic communities in household biogas digesters. Biotechnol. Biofuels.

[20] Li Y., Liu C., Wachemo A.C., Li X. (2018). Effects of liquid fraction of digestate recirculation on system performance and microbial community structure during serial anaerobic digestion of completely stirred tank reactors for corn stover. Energy.

[21] Gottardo M., Micolucci F., Bolzonella D., Uellendahl H., Pavan P. (2017). Pilot scale fermentation coupled with anaerobic digestion of food waste—Effect of dynamic digestate recirculation. Renew. Energy.

[22] Wang X., Chen G., Zhou S., Li J., Zhao X. (2019). Study on Semi-Continuous Anaerobic Fermentation of Straw Based on Total Reflux of Biogas Slurry. Chin. J. Anhui Norm. Univ. (Nat. Sci.).

[23] Sen L. (2018). Study on the Effect of the Reflux of Biogas Slurry on the Anaerobic Fermentation Characteristics of Corn Straw. Master’s Thesis.

[24] APHA (2012). Standard Methods for the Examination of Water and Wastewater.

[25] Zhong M., Duan N., Lin C., Zhang D., Liang S., Sun H. (2016). Effects of Organic Loading Rate and Additive on Corn Stalk Anaerobic Digestion. J. Biobased Mater. Bioenergy.

[26] Yang J., Wang D., Luo Z., Zeng W. (2019). Influence of reflux ratio on the anaerobic digestion of pig manure in leach beds coupled with continuous stirred tank reactors. Waste Manag..

[27] Duan N., Lin C., Su S. (2011). Analysis of pH Control Measures of Straw Anaerobic Digestion system. J. Yunnan Norm. Univ..

[28] Gulhane M., Pandit P., Khardenavis A., Singh D., Purohit H. (2017). Study of microbial community plasticity for anaerobic digestion of vegetable waste in Anaerobic Bafflfled Reactor. Renew. Energy.

[29] Zhu G.F., Li J.Z., Wu P., Jin H.Z., Wang Z. (2008). The performance and phase separated characteristics of an anaerobic bafflfled reactor treating soybean protein processing wastewater. Bioresour. Technol..

[30] Yuan Y., Wen H., Huang X., Li X., Liu X., Li D. (2014). Biogas production usingcornstalks and prokaryotic community composition. Chin. J. Chem. Eng..

[31] Jing N., Mingdian Z., Xiaofang P., Chunxing L., Nan L., Tao W., Guanjing C., Ruming W., Junjie L., Gefu Z. (2019). Simultaneous biogas and biogas slurry production from co-digestion of pig manure and corn straw: Performance optimization and microbial community shift. Bioresour. Technol..

[32] Bassani I., Kougias P.G., Treu L., Porté H., Campanaro S., Angelidaki I. (2017). Optimization of hydrogen dispersion in thermophilic up-flow reactors for ex situ biogas upgrading. Bioresour. Technol..

[33] Girma M.D., Freya M., James W.A., Daniela P., Markus G., Frank K., Lund N.J., Anders F. (2017). Exogenous addition of H_2_ for an in situ biogas upgrading through biological reduction of carbon dioxide into methane. Waste Manag..

[34] Bassani I., Kougias P.G., Treu L., Angelidaki I. (2015). Biogas Upgrading via Hydrogenotrophic Methanogenesis in Two-Stage Continuous Stirred Tank Reactors at Mesophilic and Thermophilic Conditions. Environ. Sci. Ecotechnol..

[35] Lee B., Park J.-G., Shin W.-B., Tian D.-J., Jun H.-B. (2017). Microbial communities change in an anaerobic digestion after application of microbial electrolysis cells. Bioresour. Technol..

[36] Han W., Yifeng Z., Irini A. (2016). Ammonia inhibition on hydrogen enriched anaerobic digestion of manure under mesophilic and thermophilic conditions. Water Res..

[37] Zhang Y.P., Chen G.Y., Hei K.L., Yang Y.F., Xu C.Y., Chang Z.Z. (2017). Effects of Full Continuous Reflux of Biogas Slurry on Characteristics of Rice Straw Anaerobic Digestion. J. Ecol. Rural Environ..

[38] Liu T., Sung S. (2002). Ammonia inhibition on thermophilic aceticlastic methanogens. Water Sci. Technol..

[39] Guo X., Wang C., Sun F., Zhu W., Wu W. (2014). A comparison of microbial characteristics between the thermophilic and mesophilic anaerobic digesters exposed to elevated food waste loadings. Bioresour. Technol..

[40] Ma X., Yu M., Song N., Xu B., Gao M., Wu C., Wang Q. (2020). Effect of ethanol pre-fermentation on organic load rate and stability of semi-continuous anaerobic digestion of food waste. Bioresour. Technol..

[41] Hui Z., Ming G., Miao Y., Wenyu Z., Shuang Z., Chuanfu W., Yukihiro T., Qunhui W. (2020). Methane production from food waste via mesophilic anaerobic digestion with ethanol pre-fermentation: Methanogenic pathway and microbial community analyses. Bioresour. Technol..

[42] Tian G., Zhang W., Dong M., Yang B., Zhu R., Yin F., Zhao X., Wang Y., Xiao W., Wang Q. (2017). Metabolic pathway analysis based on high-throughput sequencing in a batch biogas production process. Energy.

[43] Ros M., Filho J.d.S.O., Murcia M.D.P., Bustamante M.A., Moral R., Coll M.D., Santisima-Trinidad A.B.L., Pascual J.A. (2017). Mesophilic anaerobic digestion of pig slurry and fruit and vegetable waste: Dissection of the microbial community structure. J. Clean. Prod..

